# Diurnal physiological and immunological responses to a 10-km run in highly trained athletes in an environmentally controlled condition of 6 °C

**DOI:** 10.1007/s00421-016-3489-5

**Published:** 2016-11-09

**Authors:** Boukhemis Boukelia, M. C. Fogarty, R. C. R. Davison, G. D. Florida-James

**Affiliations:** 1 School of Applied Sciences, Edinburgh Napier University, Sighthill Campus, Sighthill Court, Edinburgh, EH11 4BN UK; 2 Department of Sport, Health and Exercise Science, University of Hull, Cottingham Road, Kingston-upon-Hull, UK; 3 Institute of Clinical Exercise and Health Science, University of the West of Scotland, Paisley, UK

**Keywords:** Immunological markers, CC16, Diurnal variation

## Abstract

**Purpose:**

The Clara cell protein CC16, secreted from Clara cells in the lung, is discussed as a potential biomarker for toxic effects on the airways. An increased concentration of CC16 in serum may be caused by increased permeability of the lungs. To investigate the changes in P-CC16 in response to an intense exercise bout performed at different times of day (9 am and 4 pm) of highly trained individuals.

**Method:**

Using a crossover randomized design, 8 runners (mean *V*O_2max_ 71 ml kg^−1^ min^−1^, SD 6) performed a 10-km time trial run, at 9 am and 4 pm, in an environmental chamber set at 6 °C. Lung function tests and blood sampling occurred at baseline, immediately post and 1 h post time trial.

**Result:**

Diurnal differences (*P* < 0.05) were found for blood neutrophil and lymphocyte counts; with higher values at 4 pm. P-CC16 was higher at the pre- and post-trial time point at 9 am compared to 4 pm. Lung function was not different between or within trials.

**Conclusion:**

Morning trial in cold condition caused more physiological strain compared to the same trial in the evening. However, this extra stress caused by zeitgebers could be a useful strategy for athletes, coaches, and general population to improve their running performance and protect their health in cold conditions in the long-term plan.

## Introduction

Highly trained athletes can be repeatedly exposed to cold air, inhalant irritants and allergens during training and racing (Helenius et al. 2005). This exposure can result in airway epithelial injury (Bolger et al. 2011), as a result of dehydration and physical stress applied to the airways during maximum effort in extreme environmental conditions (Bolger et al. 2011). Wilber et al. (2000) reported that for the seven sports evaluated on the 1998 USA Winter Olympic Team, 23% of the athletes demonstrated airway inflammation.

Several studies have reported that the risk of upper respiratory tract infection (URTI) is elevated during periods of heavy training and in the period of one to two weeks following participation in endurance races (Nieman et al. 1991; Foster 1998). Moreover, the upper respiratory tract, specifically the nasal cavity, is the first target of defence against ambient cold and irritants. Therefore, the nasal cavity represents an ideal site for the development of biomarkers of events related to airborne exposures. Airway inflammation can be detected by an increase in inflammatory markers, such as neutrophils, macrophages, inflammatory cytokines and Clara cell protein (CC16) (Bonsignore et al. 2001). CC16 is a biomarker of airway respiratory stress (Gomes et al. 2010). This protein, initially described in the epithelium of the tracheobronchial tree as a secretory product from non-ciliated Clara cells, diffuses passively from the respiratory tract into plasma and is excreted via the urinary tract (Bernard et al. 1997).

The functions of Clara cells are mainly oriented to the protection of the respiratory tract, decreasing inflammation of the airways and protecting the respiratory tract against oxidative stress (Gomes et al. 2010). Also, CC16 found in plasma has been shown to be a sensitive marker for the early detection of increases in the permeability of the lung epithelial barrier (Hermans and Bernard 1999). To date, no studies have looked at the relationship between CC16, circadian rhythmicity and sports performance. However, a limited number of studies look at the diurnal variation in CC16 within a healthy population (Helleday et al. 2006; Andersson et al. 2007). Andersson et al.’s (2007) finding was in agreement with Helleday et al. (2006) that showed a decrease in serum CC16 concentration during the daytime.

The novelty of this study is that the participants were high-level runners, tested at two different times of the day in a cold environmental condition. An array of immunological and physiological data analysed offers an important overview on the effect of exercising at a distinct time of day in cold condition. It is relevant for coaches and athletes to be aware of how the time of day can affect various physiological and immunological responses and running performance. This knowledge can allow for improved tailoring of training programs and specific activities to the time of day that generates maximum effectiveness. Furthermore, understanding the diurnal cycle of athlete performance could also be applied to testing sessions and competition times.

## Materials and methods

Eight male endurance runners (mean ± SD 32 ± 5 years, 71 ± 6 mlO_2_ kg^−1^ min^−1^, 69 ± 4 kg and 178 ± 5.7 cm) took part in this study. The participants were asked to complete a general medical history questionnaire and a specific medical questionnaire. All participants provided fully informed written consent before engaging with the experiment and were free to withdraw at any stage.

### Preliminary measurements

*V*O_2max_ test was conducted as described by Gomes et al. (2011), using online gas analysis (CPX MedGraphics, Oldham, UK); the *V*O_2max_ was started at an initial speed of 10 km h^−1^ with 0% gradient. Every 3 min, the treadmill speed was increased by 3 km h^−1^ until achieving a maximum speed of 16 km h^−1^. At this stage and after running 3 min, the treadmill gradient increased by 2.5% every minute until the runner reached maximum fatigue.

### Exercise

The exercise protocol consisted of a 10-km time trial run on a treadmill (Woodway, ergo ELG 55, Weil am Rhein, Germany), where the athletes had to complete the distance in the shortest amount of time. Two exercise trials were performed in a randomized order, one at 9 am and the other at 4 pm, with at least a 48-h interval between the trials. The trials were performed in an environmental chamber (Weis-Gallenkamp, UK) where the temperature was controlled at 6 °C with 60% relative humidity (the average British winter temperature 4.4 °C, http://metoffice.gov.uk). During the run, subjective ‘Ratings of Perceived Exertion’ (RPE), on a scale of 6–20 (Borg, 1998), heart rate (Polar Electro, Finland), and running speed were recorded at the end of each km. The athletes had free control of their running speed, without having access to the value of the speed. Also, they were allowed water ad libitum throughout the trial and were advised to refrain from intense physical activity 24 h prior to the exercise trials.

### Sample collection

Lung function tests and blood sample collection were conducted pre, post and 1 h post-trial. Lung function tests (FVC, FEV1, FEV1R, PEF, and FEF25-75) were measured using a spirometer (Compact II: Type C, Vitalograph Ltd., UK). The participants performed the test three times and the best values were recorded.

Blood samples were collected in 6-ml vacuum tubes containing EDTA (Becton–Dickinson, Oxford, UK). The samples were collected by venepuncture from a prominent antecubital vein. Cell counts and differentiation were performed with the hematoanalyser (Sysmex, xs 1000i.USA). The samples were then centrifuged for 20 min at 3000*g* at 4 °C (Mistral 2000R, Sanyo, Leicester, UK) and the plasma was aliquoted into eppendorfs (500 ml) and immediately stored at −80 °C until further analysis.

### Biochemical analysis

Plasma CC16 (P-CC16) was measured using commercially available enzyme-linked immunosorbent assay (ELISA) kits (R&D Systems, Abingdon, UK) in accordance with the manufacturer’s instructions. Also, plasma volume changes (dehydration) were calculated using the method as described in Dill and Costill (1974).

### Statistical analysis

The method of Altman (1998) was used to determine the number of subjects required for this study. Data were analysed using parametric statistics following confirmation of normal data distribution by Shapiro–Wilks’ tests. The effects of diurnal variation on physiological and biochemical measures were determined using a two-way repeated-measures ANOVA incorporating one within- (*state*: pre vs. post vs. 1 h post-trial) and one between-subjects (*group*: 9 am vs. 4 pm) factor. Statistical analyses were conducted using Minitab statistical software version 15 (Minitab Inc., UK); the alpha level was established at *P* < 0.05 and all values are reported as mean ± SD unless otherwise stated.

## Results

**Fig. 1:**
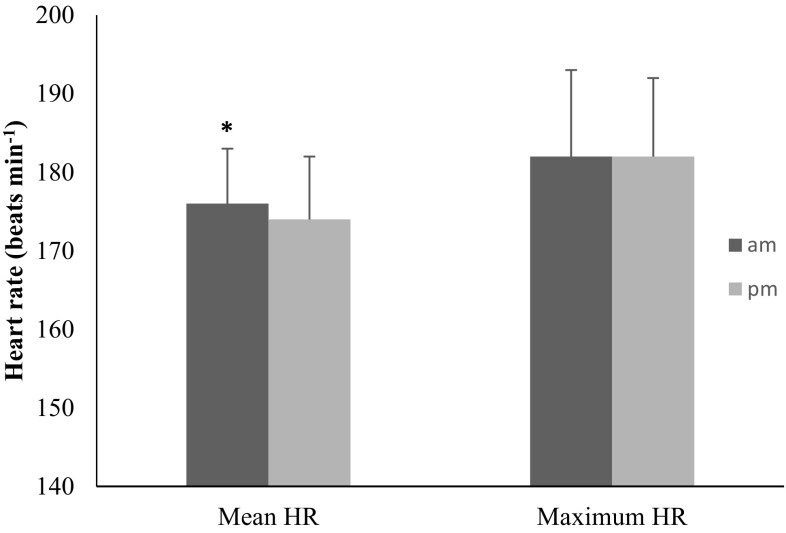
The diurnal variation in mean HR (mean ± SD). *Significant diurnal variation at the identified time point

There was no difference in the athlete’s performance time in the two conditions (Table 1). During exercise, HR demonstrated a diurnal variation (Fig. 1b), with significantly lower values (*P* < 0.05) at 4 pm compared to 9 am: were the mean HR difference was observed from the first km to 9 km. The resting and maximum at 10 km point HR values, however, were not different.

**Table 1:** Mean RPE and mean running time diurnal variation

	9 am	4 pm
Mean time	32:59:51 ± 0:09	32:58:20 ± 0:10
Mean speed	17.6 ± 0.75	17.8 ± 0.61

* Diurnal difference was observed with the variables. Values are mean ± SD

## Diurnal variation in P-CC16, WBC, neutrophil and lymphocyte

**Fig. 2:**
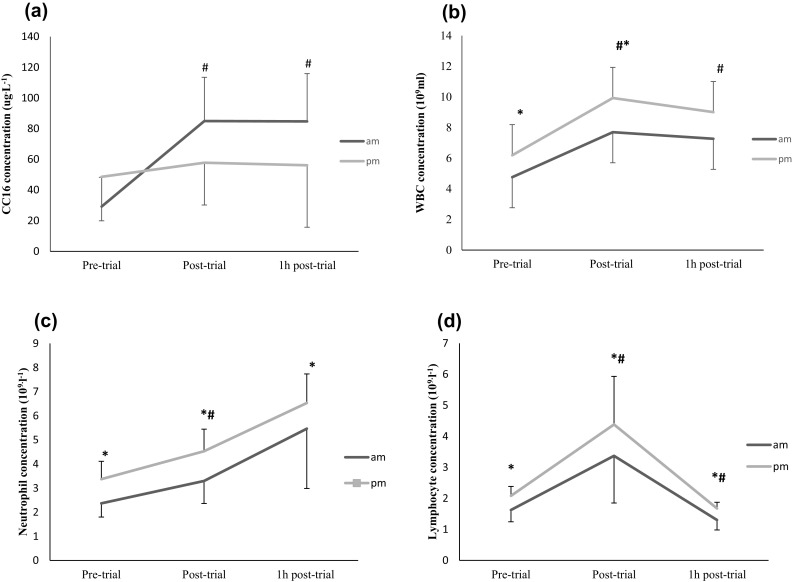
**a** Concentration of CC16 in plasma. **b** WBC, **c** neutrophil, and **d** lymphocyte concentration and circadian variation at 9 am and 4 pm (*P* < 0.05). *Significant diurnal variation at the identified time point. ^#^Significant difference between identified timed trials

The results show that neither rest nor zeitgeber has an effect on P-CC16 in participant’s diurnal variation (Fig. 2a). P-CC16 at 9 am shows a significant difference between time point trials (*F*_2,12_ = 3.97, *P* < 0.001, partial *η* ^2^ = 0.4) with Bonferroni-adjusted, post hoc test revealing that the P-CC16 has significantly changed pre to post and post to 1 h post-trial (*P* = 0.05 and *P* = 0.05) (Fig. 2a). A significant diurnal difference (*P* < 0.05) was found in blood neutrophil counts, with higher counts at 4 pm compared to at 9 am at the three measured time intervals (Fig. 2b). Likewise, a significant diurnal difference in blood lymphocyte count was also observed with greater counts at 4 pm compared to 9 am at the three time points (Fig. 2c).

No differences were found between trials or time points (*P* > 0.05) for the tested lung functions (Table 2).

**Table 2:** Lung function parameters in runners at pre-, post- and 1 h post-exercise at both times of the day: 9 am and 4 pm

	Pre-trial		Post-trial		1 h post-trial	
	9 am	4 pm	9 am	4 pm	9 am	4 pm
FVC	5.63 ± 0.91	5.59 ± 0.82	5.47 ± 0.88	5.31 ± 1.04	5.32 ± 0.88	5.77 ± 0.87
FEV_1_	4.53 ± 0.90	4.46 ± 0.77	4.56 ± 0.89	4.39 ± 1.02	4.60 ± 1.02	4.70 ± 0.84
FEV1R	0.81 ± 0.11	0.80 ± 0.09	0.83 ± 0.13	0.83 ± 0.10	0.81 ± 0.10	0.81 ± 0.08
PEF	642 ± 166	647 ± 120	604 ± 139	640 ± 130	467 ± 262	649 ± 149
FEF_25–75_	4.10 ± 1.84	4.17 ± 1.32	4.18 ± 1.75	4.48 ± 1.68	4.49 ± 1.89	4.54 ± 1.39

Forced vital capacity (FVC), forced expiratory volume in 1 s (FEV1), forced expiratory volume in 1 s ratio (FEV1R), forced expiratory flow (FEF) and forced expiratory flow 25–75% (FEF_25–75_) show no diurnal variation during the trials

## Diurnal variation in red blood cell variables and platelet

The mean concentration of RBC, HGB, HCT and PLT showed both no diurnal variation and no significant difference between trials times point (Table 3).

**Table 3:** Diurnal variation in total red blood cells (RBC), haemoglobin (HGB), haematocrit (HCT) and platelet (PLT), values are mean ± SD (10^9^ l^−1^)

	Pre-trial			Post-trial			1 h post-trial		
	9 am	4 pm	%Δ	9 am	4 pm	%Δ	9 am	4 pm	%Δ
RBC	5.35 ± 0.39	4.87 ± 0.38	10	5.23 ± 0.49	5.04 ± 0.17	4	4.88 ± 0.27	4.92 ± 0.21	<1
HGB	16.2 ± 1.25	14.8 ± 0.72	9	15.9 ± 1.64	15.4 ± 0.59	3	14.9 ± 0.74	15.0 ± 0.59	<1
HCT	48.2 ± 3.51	44.0 ± 2.08	9	47.4 ± 4.91	45.7 ± 1.81	4	44.1 ± 1.89	44.5 ± 1.40	<1
PLT	168 ± 12	216 ± 35	29	288 ± 44	275 ± 49	20	174 ± 24	229 ± 40	32

Values are mean ± SD

## Diurnal variation in plasma volume (dehydration)

Plasma volume was 53.8 + 5.32 and 52.2 ± 4.95 ml at 9 am and 4 pm, respectively. An increase of 0.26 g 100 ml^−1^ in HGB concentration was reported at 9 am and an increase by 0.06 g 100 ml^−1^ at 4 pm. There was no diurnal variation observed in plasma volume dehydration (*P* = 0.80); the plasma volume was 3% lower in the evening compared to the morning.

## Discussion

This study examined the effect of the time of the day on 10-km treadmill running at 6 °C. This represents the typical mean winter conditions within the UK and Western Europe (http://metoffice.gov.uk). The physiological parameter of HR was more affected by zeitgeber in the morning compared to the evening time-point. However, no statistical differences were found for running performance between these time points. Interestingly, a diurnal variation was observed for neutrophil and lymphocyte counts.

Athletes’ HR was significantly higher in the morning trial, which indicates that participants in the morning showed higher physical demand during this trial compared to the evening. Also, from a clinical perspective, the morning HR may be a cause for concern for cardiovascular patients that are attempting to practise exercise in the morning in cold condition.

RBC total counts, HCT, HGB, PLT, lung function parameters and plasma volume (dehydration) did not show either a diurnal significant difference between morning and evening trials or at any time point during the trials. This result corroborates with Simpson et al. (2007) where RBC, HCT and HGB did not change in fourteen London marathon finishers.

To the best to our knowledge, this is the first study investigating the diurnal difference on P-CC16 in highly trained runners (>70 *V*O_2max_). The concentration of P-CC16 was found to not be affected by the time of the day. This is in disagreement with the published findings of Helleday et al. (2006) who reported a decrease in P-CC16 concentration during daytime, with significant drop between 11.30 am and 10 pm. In contrast, Andersson et al. (2007) found an increase in the concentration of CC16 in urine throughout the day, with significant increase 20 h after exposure to wood smoke. Nevertheless, this protein can be used as a specific biomarker of the airway epithelium integrity and an approach to estimate the degree of lung epithelial injury (Gomes et al. 2011), thus identifying respiratory tract inflammation which is the most common medical condition affecting both highly trained and elite athletes, in particular, those participating in endurance events (Bermon 2007). It is known that environmental conditions impact on the degree of airway epithelial disruption during high-level exercise (Bolger et al. 2011 **)**. When cold air was inhaled, there was a rise in CC16. Moreover, in our study, the degree of leakage of CC16 could be directed related to the cold environment; this corresponds with Bolger et al. (2011) where after 8 min of exercise in dry air causing an epithelial injury in the subjects taking part in the study.

In the present study, we did not aim to assess reasons for the diurnal variation in the P-CC16 concentrations, and nothing is known about the underlying mechanisms here. We can speculate on a few possible explanations for the higher phase response which was more pronounced in the morning trial. According to McAuley and Matthay (2009), a decrease in P-CC16 concentration could reflect the differentiation of distal lung epithelial cells into alveolar epithelial cells as part of the repair processes. Another explanation could be a difference in transepithelial leakage due to cyclic changes in the tightness of the epithelial tight junctions. Addressing in detail the mechanisms behind variation in P-CC16 conventions is an important aspect to consider for future research.

A significant diurnal difference was observed in total white blood cells, neutrophil and lymphocyte counts. In contrast, monocytes, basophil and eosinophil are not affected by circadian rhythm. Monocytes show a significance difference pre- to post at 4 pm; whereas eosinophil and basophil were significant at both times of the day increasing from pre- to post-trial. As expected, the exercise bout was sufficient to promote a change in lymphocyte counts increasing immediately post-trial compared to those pre-trial or at 1 h post-trial at both times of the day. This result supports the findings of other published work: that intense exercise suppresses the immune system (Simpson et al. 2007; Walsh et al. 2011). Similarly, as anticipated, neutrophil counts significantly increased 1 h post-trial compared to pre- and post-trial at both times of the day. This post-trial increase in neutrophils and lymphocytes is considered to result in an “open window” of decreased host protection, which can last between 3 and 72 h and represents a vulnerable time period for the individual contracting and developing an infection (Nieman and Bishop 2006). The higher neutrophil, lymphocyte counts at 4 pm compared to 9 am is possibly due to the athletes’ daily winter exposure and the cold environment. Another possibility may be the influence of the endocrine hormones as seen with regards to other immune cells (Dimitrov et al. 2009). Both chemical and nutritional interventions have been recommended for athletes to minimize potential negative changes in immunity during periods of intensity training.

It is well known that circadian rhythms cause narrow bronchial calibre in humans in the early hours of the morning (4 am); this may lead to high immunological morning suppression. This article has provided evidence to support the hypothesis that strenuous exercise alters the immune system in well-trained athletes; this dysfunction has reported to lead to an URTI (Nieman et al. 1991; Foster 1998). This study found that lung inflammation is more severe in the morning as evidenced by higher CC16. Previous studies have found links between CC16 and epithelium injury, as well as URTI and epithelial injury (Nieman et al. 1991; Foster 1998). This study did not investigate URTI, however, future research should consider the possibility of time of day variation in URTI in line with lung inflammation and epithelial injury that is greater in the mornings. Furthermore, neutrophils phase response was higher at 9 am when epithelial damage was also reported to be associated with mobilisation of neutrophils in the airway (Yoshihara et al. 2006). In contrast, other immunological parameters such as lymphocytes occur in the afternoon, were this variable can be linked also to URTI.

It can be concluded that the morning trial in a cold condition caused more physiological and immunological strain compared to the same trial in the evening. However, this extra stress caused by zeitgebers could be a useful strategy for athletes, coaches, and general population to improve running performance (fitness) in cold condition in the long-term plan.

## Perspective

The athletes in this study were highly fit (mean *V*O_2max_ <71 ml kg^−1^ min^−1^), training, mostly, twice-a-day during the pre-race season. However, exercising at high intensity for a prolonged period of time with insufficient recovery can reduce the body’s ability to fight infection at any time of the day. Running performance was not affected despite the time of the day, most likely due to the subjects’ standard (S-shape). Athletes, coaches and event organisers can program their race events or trainings to target the time when athletes perform best. There is a need to look at psychological factors that may affect running performance in elite athletes. Furthermore, addressing in detail the mechanisms behind variation in P-CC16 conventions is an important aspect to consider for future research.

## Abbreviations

CC16: Clara cell 16
ELISA: Enzyme-linked immunosorbent assay
FEF: Forced expiratory flow
FEF_25–75_: Forced expiratory flow 25–75%
FEV1: Forced expiratory volume in 1 s
FEV1R: Forced expiratory volume in 1 s ratio
FVC: Forced vital capacity
HCT: Haematocrit
HGB: Haemaglobin
RBC: Red blood cell
PLT: Platelet
WBC: White blood cells
